# Long-term endocrine outcomes and quality of life in paediatric and young chronic myeloid leukaemia patients on tyrosine kinase inhibitor therapy: a prospective study from India

**DOI:** 10.3389/fonc.2025.1598104

**Published:** 2025-07-08

**Authors:** Rajiv Mohan, Chitrakshi Nagpal, Swasthik Upadhya, Akash Kumar, Rajesh Khadgawat, Sameer Bakhshi, Deepam Pushpam

**Affiliations:** ^1^ Department of Medical Oncology, Dr. B. R. Ambedkar Institute Rotary Cancer Hospital, All India Institute of Medical Sciences, New Delhi, India; ^2^ Department of Medical Oncology, National Cancer Institute, All India Institute of Medical Sciences, Jhajjar, Haryana, India; ^3^ Department of Endocrinology and Metabolism, All India Institute of Medical Sciences, New Delhi, India

**Keywords:** endocrine effects, quality of life, CML, paediatric, young, TKI

## Abstract

**Background:**

Chronic myeloid leukaemia (CML) is rare in paediatric population, approximately 2–3% of leukaemia cases in children and 9% in adolescents. The introduction of tyrosine kinase inhibitors (TKIs) has significantly improved outcomes, but long-term use may lead to adverse endocrine effects and impact quality of life (QoL).

**Methods:**

This prospective cross-sectional study evaluated 82 paediatric and young adult CML patients who were started on TKI therapy at <18 years of age. 71 patients were on TKI for >2 years and 11 patients were initiated on TKI during the study. Anthropometric measurements, Tanner staging, and laboratory parameters assessing endocrine function, bone health, glucose and lipid metabolism were evaluated. QoL was assessed with validated instruments.

**Results:**

Median duration of TKI exposure was 7.5 years. Short stature was observed in 15% (11/71) of patients. Among those who were prepubertal at TKI initiation, 26.5% (9/34) were found to have short stature. Low Bone mineral density was found in 35·2% (24/68). Vitamin D deficiency was found in 84% and subclinical hypothyroidism in 9·8% (7/71). Two patients had poor sperm quality. One male patient had infertility. There was less impact on glucose, lipid profile, and adrenal function. In the QoL analysis, social functioning was affected among the functional scales, while financial difficulties, appetite loss, and fatigue were impacted in the symptom scales. Overall, QoL scores for physical, psychosocial, and school functioning remained well-preserved, but longer TKI treatment duration was associated to lower scores in these domains.

**Discussion:**

This comprehensive evaluation highlights the need for close monitoring of BMD in patients who are on long-term TKI therapy. Evaluation of all other endocrine functions can be done as clinically indicated.

## Introduction

Chronic myeloid leukaemia (CML) is rare in paediatric population, comprising approximately 2–3% of leukaemia cases in children and around 9% in adolescents (1). The introduction of tyrosine kinase inhibitors (TKIs) has transformed the treatment landscape of CML, significantly improving outcomes without the need for allogeneic stem cell transplantation (2). CML in children may behave differently than adults (3) and unlike adults, paediatric patients with CML will likely require multiple decades of TKI treatment, which may lead to additional long-term off target adverse effects. Growing children may be possibly susceptible to a different side effect profile than adult patients. Previous research has predominantly focused on the effect of TKI on growth and development, with retrospective studies (4, 5) and meta-analysis (6) shedding light on this aspect. However, information regarding effects on other crucial aspects such as bone health (7), thyroid function (8), sexual development (9), glucose and lipid metabolism (10, 11), quality of life (QoL) remains largely unexplored. Therefore, this study aims to analyse the long term off- target adverse effects of TKI on endocrine functions and QoL in CML patients who were diagnosed at <18 years of age.

The primary objective was to study the endocrine and metabolic effects of TKIs which included bone mineral metabolism, thyroid function, gonadal function, glucose, lipid metabolism, and growth in CML patients diagnosed at age <18 years and had been on TKI treatment for at least 2 years. The secondary objectives were to study effect of TKI on endocrine function in CML patients who were newly diagnosed and initiated TKI at age <18 years during the study regardless of duration of TKI, and to study the effect of TKI on QoL in entire cohort.

## Methods

### Study design

In this prospective observational study, patients diagnosed with CML who were registered over 2 decades from January 2004 to January 2024 at the Department of Medical Oncology at All India Institute of Medical Sciences, New Delhi and National Cancer Institute Jhajjar were included.

### Study conduct

Patients with CML who were started on TKI at age less than 18 years and had been on TKI treatment for at least 2 years, or newly diagnosed CML patients < 18 years, who started TKI during the study period were included in the study, regardless of the duration of TKI. Patients who were treated with chemotherapy regimens or who underwent bone marrow transplant were excluded from the study. Patients with major cognitive deficit, psychological problems hampering self-reported outcome evaluation were excluded from QoL assessment. Informed Consent was taken. The study was approved by institutional ethics committee.

Height (in centimetre) and weight (in kilogram) were measured by stadiometer and electronic weighing scale respectively. Body mass index (BMI) was calculated from height and weight. World Health Organisation (WHO) multicentre growth reference study group curves were utilized as reference (12). Height less than two standard deviations (SDs) for age and gender- matched reference or the inability to attain the mid-parental height (MPH) was considered as short stature (13). Growth hormone (GH) and insulin like growth factor-1 (IGF-1) levels were measured for all the patients, and were compared with age wise normal levels (14). In patients with short stature, bone age was assessed using X-ray of wrist and hand. Tanner stage was assessed, and testicular volume was measured using Prader’s orchidometer. Breast examination was done to evaluate for presence of gynecomastia. Pubertal age was defined as being younger than 9 years for girls or 11 years for boys (15). The absence of physical signs of sexual maturity in boys and girls by the age of 14 and 13 years, respectively, were defined as delayed puberty.

Sex hormonal analysis including follicular stimulating hormone (FSH), luteinising hormone (LH), and testosterone in males, oestrogen in females were done. Bone health was assessed using serum calcium, phosphorus, vitamin D3 and intact parathyroid hormone levels (iPTH), x-ray lumbo-sacral spine and dual-energy X-ray absorptiometry (DEXA) scans. Bone mineral density (BMD) Z-score between −1·0 and −2·5 was referred to as osteopenia, whereas a Z-score of −2·5 or less was defined as osteoporosis (16). Vitamin D deficiency and insufficiency were defined as 25-hydroxyvitamin D levels less than 20 and 30 ng/mL respectively (17).

Thyroid function was assessed by T4, and thyroid stimulating hormone (TSH). Adrenal function was assessed by adrenocorticotrophic hormone (ACTH) and 8AM cortisol. Fasting blood glucose, oral glucose tolerance test with 75-gram glucose and HbA1c were assessed (see **Supplementary Table 1**). Diabetes was diagnosed based on American Diabetes Association (ADA) guidelines (18). Homeostasis Model Assessment - Insulin Resistance (HOMA-IR) index was calculated from fasting insulin and fasting blood sugar.

All the mentioned assessments of endocrine function were done in entire cohort at initiation of study. For patients who were newly diagnosed during the study, the assessment was repeated at 6 monthly intervals. Each patient was evaluated at a single point in time, and the timing of this evaluation varied depending on the duration of TKI therapy.

QoL was assessed by EORTC QLQ-C30 questionnaire (19) for patients aged >18years and by PedsQL generic score (20) in patients with age <25years. For reference EORTC reference values manual (2^nd^ edition) was used for QLQ-C30 scoring. A score difference of >5 was considered significant.

### Statistical analysis

Data analysis was done using SPSS software (*IBM Corp. (2020). IBM SPSS Statistics for Windows (Version 27·0). IBM Corp.*). The baseline characteristics were represented using descriptive statistics. Categorical data was expressed in the form of percentages and continuous variables as median. The difference in categorical variables between the groups was analysed using chi-square test. The difference in continuous variables was analysed using Mann-Whitney U test. HRQoL was compared between the patients who are on TKI >2 years and patients who started on TKI during the study using unpaired T test. Correlation with type of TKI and duration of TKI with QoL was done using linear regression. A pre-specified p-value of less than 0·05 was considered as significant.

## Results

A total of 82 patients of CML with age <18 years at the time of TKI initiation were enrolled in the study. Seventy-one (86·6%) patients were already on TKI for >2 years and 11 (13·4%) were started on TKI during the study. Seventy-eight (95·1%), three (3·7%) and one (1·2%) patients presented in the chronic (CP), accelerated (AP) and blastic phase (BP), respectively. The median ages of the patients at the time of diagnosis and at the time of study were 12 (2-17) and 21 (5-36) years, respectively. Of 82 patients, 66 (80·5%) were male. Median duration of exposure to TKI in the whole cohort was 7.5 years. Thirty-four (41·5%) patients were prepubertal and 48 (58·5%) were post pubertal at the time of diagnosis. Twenty-eight (34·2%) patients were <18 years and 30 (36·6%) patients were ≥25 years of age at the time of evaluation. (**Table 1**).

**Table 1:** Baseline characteristics at presentation.

Clinical features at baseline	Mean (SD) or n (%)
Age at diagnosis (n = 82)	12·1 (± 3·5)
Age at the time of study (n = 82)	20·6 (± 6·5)
WBC (10^9^/L) (n=75)	301 (± 550)
Platelets (10^9^/L) (n=75)	452 (± 462)
Spleen (cm) (n=73)	10·1 (± 5·8)
ELTS score (n=73)	Low – 25 (34·2)
	Intermediate – 33 (45·2)
	High-15 (20·5)
1^st^ line TKI, n (%)	Imatinib-80 (97·5) Dasatinib-2 (2·4)
Current TKI, n (%)	Imatinib - 71 (86·5) Dasatinib – 10 (12·2) Nilotinib - 1 (1·2%)
Median duration of TKI at the time of study Median (Range)	7·5 years [0·3-22]
Patients in DMR at the time of study, n (%)	50 (70·4%)
Puberty status at TKI initiation - n (%)	Prepubertal: 34 (41·5%) Post-pubertal: 48 (58·5%)

ELTS-EUTOS long term survival score; DMR, deep molecular response; SD, Standard deviation; TKI, tyrosine kinase inhibitor; WBC, white blood cell count.

Eighty (97·6%) patients received imatinib and 2 (2·4%) received dasatinib as first-line. Nine (11·0%) patients switched to second-generation TKIs (dasatinib-8, nilotinib-1) due to lack of adequate response or loss of molecular remission. At the time of analysis, 71 (86·6%), 10 (12·2%) and 1 (1·2%) patients were on imatinib, dasatinib, and nilotinib respectively. Median duration of TKI exposure was 7·5 (0·3-22) years. Fifty (70·4%) patients had deep molecular response (DMR), and 44 out of 64 evaluated patients had major molecular response (MMR).

### Patients on TKI for more than two years

#### BMI

BMI was normal in 39 (47·6%) patients. Thirty-two patients (44%) had an abnormal BMI; 21 (25·6%) were underweight, 11 (13·4%) were overweight, and 1 (1·2%) was obese. Patients with an abnormal BMI had a younger age at the time of TKI initiation and were more likely to be pre-pubertal compared to the ones with a normal BMI. (**Supplementary Table 3**). Across BMI categories, age at TKI initiation, age at time of study, and duration of TKI therapy differed significantly (all p < 0.005, Kruskal-Wallis test). Additionally, pubertal status at TKI initiation was significantly associated with BMI category (chi-squared = 8.37, p = 0.015).

#### Bone health, parathyroid and vit D

DEXA scan was done in 68 patients, out of which low BMD was seen in 24 (35·2%) patients with a median duration of exposure to TKI of 10·5 years (3·7-22). Of these, 15 (62·5%) and 9 (37·5%) patients had osteopenia and osteoporosis, respectively (**Table 2**). Median Z-scores for patients with normal BMD, osteopenia and osteoporosis were 0·6 (0 to -1.8·9), 1·53 (-1·1 to -2·3) and -3·0 (- 2·6 to -4·0) respectively. All patients with low BMD had subnormal levels of serum vitamin D. PTH was normal in 18(75%) patients. Calcium, phosphorous, alkaline phosphatase levels were normal in all the patients. On comparison of clinical and biochemical variables between patients with normal and low BMD (**Table 2**), no significant differences were observed in age at TKI inititation (p = 0.16), pubertal status at TKI initiation (p = 0.277), duration of TKI (p = 0.7) or proportion of patients with elevated PTH (p = 0.463). However, Vitamin D deficiency/insufficiency was significantly more frequent in patients with low BMD (p = 0.013). Nine (32·1%) patients had both short stature and low BMD.

**Table 2:** Patients with normal BMD and Low BMD.

Parameter	Normal BMD (n=44)	Low BMD (n=24)	p-value*
Median z score (range)	-0·6 (0 to 1·8)	-2·3 (-1·1 to - 4)	–
Pre-Pubertal at initiation of TKI n (%)	22 (50·0%)	9 (37·5%)	0.28^†^
Post-Pubertal at initiation of TKI n (%)	22 (50·0%)	15 (62·5%)	–
Age at TKI initiation-Mean (SD)	11·9 (± 3·9)	11·8 (± 3·6)	0·16*
Age at time of study Mean (SD)	21·2 (± 6·6)	22·8 (± 5·7)	0·39*
Duration of TKI in years Median (range)	8·5 (2·3-19·5)	10·5 (3·6 – 22·0)	0·70*
Elevated PTH n (%)	11 (25·0)	6 (25·0)	0.46^†^
Vitamin-D Insufficiency Deficiency n (%)	11 (25·0) 21 (47·7)	9 (37·5) 15 (62·5)	0.01^†^

BMD, bone mineral density; PTH, Parathyroid hormone; SD, standard deviation; TKI, tyrosine kinase inhibitor. BMD could not be done in 3/71 patients.

*Mann Whitney U test.

† Chi-square test for categorical comparisons.

Parathyroid hormone was elevated in 17 patients with all patients having subnormal vitamin D levels (**Supplementary Table 4**). Among these, Six patients(6/17) had low BMD. No significant difference was found in age at TKI initiation, duration of TKI and type of TKI between patients with normal PTH and Elevated PTH. 66 (93%) patients had Vitamin D deficiency/insufficiency.

#### Thyroid function

Seven patients had subclinical hypothyroidism (**Supplementary Table 4**). One female was diagnosed with hypothyroidism with anti TPO antibodies being negative. In the patients who were started on TKI during the study, thyroid function was normal.

#### Gonadal function

Pubertal status was normal in all except for one male patient. Infertility with semen analysis showing poor motility of sperms and a normal sperm count was found in one patient. Poor secondary sexual characters with elevated LH, FSH and normal testosterone levels were found in another patient. Semen analysis showed only few motile sperms.

LH was elevated in 19 patients (26·3%). Of these, FSH was elevated in two patients, one had low testosterone (found to have Klinefelter syndrome), the other female refused for further evaluation. In the rest of the patients with elevated LH, the secondary sexual characteristics, FSH and testosterone were normal. Three patients had gynecomastia. Menstrual cycles and hormonal profiles were normal in female patients. Out of 13 patients who are married, 12 (92·3%) had healthy children; one had infertility. Adrenal function was normal in all the patients except for one with low cortisol levels.

#### Glucose and lipid metabolism

Dysglycemia was seen in eight patients (11%). Fasting Blood glucose was elevated in four patients, HbA1C was in pre diabetic range in three patients. One patient had diabetes. The mean (± SD) HOMA IR score of the patients was 3·7 (± 3·9). Dyslipidaemia was seen in 16 patients (22·2%). LDL was in above optimal levels in four (5%) patients. Eight (11%) patients had borderline high, and six (8%) patients had high triglyceride levels. VLDL was elevated in six patients (8%).

#### Growth

Out of 71 patients who were already on TKI >2 years, 11 patients (15·5%) had short stature. **Table 3** presents a comparative analysis between patients with short stature and those with normal height. Patients with short stature had a significantly lower mean age at TKI initiation (8.7 ± 4.0 vs. 12.7 ± 3.3 years, p=0.0001) and at the time of evaluation (16.6 ± 6.3 vs. 22.9 ± 5.6 years, p=0.001) compared to those with normal height. Most of them (81.8%) had started treatment before the onset of puberty. Although the median duration of TKI therapy was shorter among those with short stature (6 vs. 10 years), the difference was not statistically significant (*p*=0.08). All patients with short stature were receiving imatinib.

**Table 3:** Patients who are on TKI > 2years with short stature Vs normal height.

Parameter	Short Stature (n= 11)	Normal height (n=60)	p-value
Prepubertal at initiation of TKI n (%)	9 (81·8)	23 (38·3)	–
Post pubertal at initiation of TKI n (%)	2 (18·0)	37(61·7)	–
Age at TKI initiation-Mean (SD)	8·7 (± 4·0)	12·7 (± 3·3)	<0.001*
Age at time of study Mean (SD)	16·6 (± 6·3)	22·9 (± 5·6)	0·001*
TKI at the time of study n (%)	Imatinib-11 (100·0)	Imatinib-53 (88·3) Dasatinib-6 (10·0) Nilotinib-1 (1·7)	0·49^#^
Median duration of TKI in years Median(range)	6 (3-16)	10 (2·3-22·0)	0·08*

SD, Standard deviation; TKI, tyrosine kinase inhibitor. *Mann Whitney test ^#^Chi-square test.

When growth outcomes were examined based on age at the time of study, short stature was notably more common in younger individuals: 8 out of 18 patients (44.4%) who were under 18 years had short stature (see **Supplementary Table 2**), compared to just 3 out of 53 patients (5.6%) aged 18 years or older. Importantly, the few older patients with short stature had not attained their expected height potential, suggesting long-lasting growth impairment. None of the patients with short stature had growth hormone deficiency, although low IGF-1 levels were found in three individuals.

### Patients started on TKI at the start of study

In 11 patients who were started on TKI during the study, one patient had low BMD and three patients had vitamin D deficiency. No other endocrine abnormalities were identified at the time of evaluation. There was no difference in height during the follow up.

### Quality of life

Quality of Life (QoL) was evaluated using the PedsQL for patients aged 25 years or younger and the EORTC QLQ-C30 for those above 18 years, with the findings summarised in **Table 4**. Considering the modest sample size and the non-normal distribution of scores, group comparisons were appropriately performed using the non-parametric Mann–Whitney U test. Among patients assessed with the PedsQL, those who had achieved a major molecular response (MMR) reported significantly better QoL (p = 0.0015), while no meaningful differences were noted based on sex, pubertal status at treatment initiation, or short stature (all p > 0.70). Notably, linear regression revealed a strong positive association between QoL and the time elapsed since diagnosis (p < 0.0001), suggesting that patients tended to report better well-being the longer they had been living with the disease. It is important to note that this reflects a cross-sectional analysis, with each patient contributing data at a single point in time. The regression coefficient indicates a gradual increase in QoL scores with each passing year after diagnosis. On the other hand, EORTC QLQ-C30 scores showed no significant relationship with these clinical variables, except for a borderline difference with pubertal status (p = 0.050), and were not significantly influenced by time since diagnosis (p = 0.548). This analysis reflects cross-sectional data; QoL was assessed at a single time point per patient. No longitudinal follow-up was performed.

**Table 4:** Comparison of quality of life scores by clinical subgroups.

Domain	PedsQL p-value	EORTC p-value	Interpretation
Sex*	0.9157	0.77	No difference
Pubertal Status*	0.7703	0.05	No difference
MMR Status*	0.0015	0.33	Significant in PedsQL
Short Stature*	0.7065	0.47	No difference
Time Since Diagnosis^#^	<0.0001	0.55	Significant only in PedsQL

*Mann–Whitney U test.

^#^ Linear regression.

## Discussion

Multiple studies on long-term effects of TKI have reported growth retardation as an important adverse effect in children who were treated with imatinib for a longer duration, but majority of them were retrospective studies (4, 5). The data regarding other endocrine effects is sparse. This study represents one of the largest cohorts of paediatric CML patients with long term follow up, providing a prospective comprehensive evaluation of the long-term side effects of TKIs on endocrine function and QoL. The median duration of TKI in our study is 7·5 years. As shown in previous studies, the majority of patients began treatment with the first-generation TKI, imatinib, due to its availability, safety profile, and the financial inaccessibility of second-generation tyrosine kinase inhibitors (2).

We found short stature in 15% of patients who were all on imatinib. The age at onset of TKI was significantly lower than those with normal height similar to studies by Moulik et al (21) and Cai et al. (22) Most of the patients were <18 years at the time of study and all patients were on imatinib. Our findings were similar to previous studies (5, 22) which showed factors like age at TKI initiation, duration of TKI has positively correlation with short stature. Growth impairment was predominantly seen in patients who started TKI in prepubertal age, which is similar to the previous studies (23, 24). Notably, there are other studies which did not find any difference in pubertal and post pubertal age of TKI initiation (4). The difference in these study results might be due to difference in definition of puberty used in different studies. Some studies (4, 24, 25) have described catchup growth after attaining puberty which could not be assessed in our study due to cross sectional nature of our study. Final height in three of our patients who were more than 18 years of age, was less than the target height potential indicating lasting effect on growth with TKI which is in similar to previous study by Rastogi et al. (26) that have shown lasting effect on height.

In patients with short stature, low IGF-1 levels were found in three cases, while growth hormone levels remained within the normal range. None of our patients required GH therapy. Normal Growth hormone levels in these patients suggest the possibility of growth hormone insensitivity as cause of short stature, which was proposed in previous studies (5). However we did not assess GH insensitivity which is a limitation of the study. The exact mechanism of growth retardation in children treated with TKIs is not known. Mechanisms such as inhibition of the activity of non-BCR-ABL1 kinases (PDGFR-β and c-KIT) resulting in disruption of the signals at the growth plate were postulated (27). In a study by Narayan et al., it was shown that imatinib results in growth failure in children with CML by disturbing the GH: IGF-1 axis. GH deficiency has also been observed with imatinib therapy (5). Our results emphasize the need for long term follow up of patients on TKI for identification of persistent effect on growth. All the previous studies have short term follow up of patients where the long-term effect on growth has not been properly assessed.

In our study, children with abnormal BMI were more likely to have started TKI therapy at a younger age and were more often prepubertal at the time of initiation. These observations were supported by statistical analysis: significant differences in age at TKI initiation, age at the time of study, and duration of TKI therapy were seen across BMI categories. Additionally, pubertal status at TKI initiation was significantly associated with BMI (p = 0.015). Taken together, these findings suggest that earlier initiation of TKI therapy and prepubertal status may influence BMI patterns over time in this patient group.

Imatinib is known to cause dysregulation of bone remodelling and mineralization (7). In our study a significant proportion (35·2%) of patients had low BMD. The mean age at the time of initiation of TKI was 11·8 years. Of these 50% were prepubertal at the time of initiation. At the time of recruitment, 79% of the participants were >18 years of age. Patients on TKIs other than imatinib were also affected. Sixty percent had vitamin D deficiency, and 24·2% patients had elevated parathyroid hormone levels. The data on effect of TKI on BMD is sparse, only case series and one retrospective study were reported (21, 24). In their previous studies, Deng et al. (28) and Moulik et al. (21) have shown prevalence of low BMD to be 42·9% and 70% respectively. Secondary hyperparathyroidism with normal BMD was reported in previous studies in adult patients (6), similarly in our study patients had elevated parathyroid hormone levels with normal and low BMD with normal calcium and phosphorous levels. Our comparison of clinical variables confirmed that Vitamin D deficiency was significantly more frequent among patients with low BMD (p = 0.013), while no significant associations were observed with pubertal status, type of TKI, or elevated PTH. Our data indicate that imatinib primarily affects bone resorption rather than osteogenesis in children and young patients, unlike in adults where it promotes bone formation and inhibits resorption, leading to lower serum calcium and phosphate levels (7).

There are only few studies regarding the effect of TKI on thyroid function, in this age group. Retrospective studies in adults have shown prevalence of both hypothyroidism and hyperthyroidism (8). An Indian study (21) has reported hypothyroidism in 7% of their patients, majority of them being less than 5 years of age. In our study 10% of patients had subclinical hypothyroidism, none of our patients were <5 years of age. These patients were kept on follow-up. Only one patient had hypothyroidism that required therapy. The median age of afore mentioned study population was 10·8 years with 15·6% patient’s underage of 5 years, which is different from our study. This might have led to the difference in the findings between the two studies. In our study two patients had poor sperm quality, and one patient had infertility which is less than that reported in study by Moulik et al. (21), which was 43%. Reduced sperm density, counts, survival rates, and activity were observed in study by Chang et al. (9) Data on the infertility is sparse, prospective studies are required to properly assess the effect.

LDL was elevated in few patients, there are no major risk factors that indicate treatment, lifestyle modifications were advised. Fasting hyperglycaemia and HbA1c in prediabetic range were observed in contrary to the prior retrospective studies in adults which have shown improvement in fasting blood glucose levels (10, 11). The mean HOMA-IR values were higher indicating impaired sensitivity to insulin. These findings are similar to study by Iurlo et al. in which diabetes/impaired fasting blood glucose were identified in 25% of the imatinib and dasatinib-treated patients, and 33% of those on nilotinib (11).

This is one of the largest prospective studies which included QoL analysis in this age group. Median QoL scores were similar to the reference scores. Although the time since diagnosis was not independently predictive of the total QoL score, it predicted various domains of PedsQL – physical, psychosocial functioning independently. There was no association of the female gender, age at diagnosis, or whether MMR was achieved or not with PedsQL. In a similar study, Zheng et al. (29) had assessed QoL in paediatric CML patients who are predominantly male children (62%) with median age of 13 years. They found that female gender and younger age at study were predictive factors of a worse HRQOL among others. In our study we did not find any association with these variables and female sex was under-represented in the cohort which may have confounded the results.

Long term follow-up of endocrine effects like growth, BMD, dysglycemia and dyslipidaemia is required. Low BMD is a significant area of concern which is to be monitored and treated appropriately. In previous studies regular follow up all for endocrine functions was recommended (30), however in our study we did not find major issues in endocrine function except for growth and low BMD. Though the prevalence of subclinical hypothyroidism and dysglycemia are slightly higher, they did not require any therapy. Our study suggests that clinical monitoring of growth and BMD is required. Evaluation of all other endocrine functions can be done as clinically indicated.

Due to rarity of the CML in children, the sample size was limited. Prospective multicentric collaboration may allow for more data collection and validation. A key limitation is the cross-sectional nature of our evaluation, which precluded real-time serial monitoring and the availability of baseline values for growth, endocrine, and quality of life parameters. Furthermore, patients were assessed at varying durations after TKI initiation, introducing some heterogeneity in exposure and outcomes. Despite these limitations, our study uniquely followed a cohort of paediatric CML patients and prospectively explores the long-term effects of TKI on endocrine system. This includes novel insights into effects of TKI on parathyroid, thyroid, gonadal function as well as glucose and lipid metabolism. Additionally, it adds valuable data on QoL of paediatric CML patients,an area with limited existing literature.

## Data Availability

The raw data supporting the conclusions of this article will be made available by the authors, without undue reservation.

## References

[1] Hijiya N Schultz KR Metzler M Millot F Suttorp M. Pediatric chronic myeloid leukemia is a unique disease that requires a different approach. Blood. (2016) 127:392–9. doi: 10.1182/blood-2015-06-648667, PMID: 26511135 PMC4915793

[2] Ganguly S mian A Chopra A Gupta R Pushpam D Bakhshi S. Real-world experience of imatinib in pediatric chronic phase chronic myeloid leukemia: a single-center experience from India. Clin Lymphoma Myeloma Leuk. (2020) 20(7):e437–44., PMID: 32247650 10.1016/j.clml.2020.02.015

[3] Pushpam D Bakhshi S. Paediatric chronic myeloid leukaemia: Is it really a different disease? Indian J Med Res. (2019) 149:600–9., PMID: 31417027 10.4103/ijmr.IJMR_331_19 PMC6702689

[4] Millot F Guilhot J Baruchel A Petit A Leblanc T Bertrand Y. Growth deceleration in children treated with imatinib for chronic myeloid leukaemia. Eur J Cancer Oxf Engl 1990. (2014) 50:3206–11. doi: 10.1016/j.ejca.2014.10.007, PMID: 25459396

[5] Narayanan KR Bansal D Walia R Sachdeva N Bhansali A Varma N. Growth failure in children with chronic myeloid leukemia receiving imatinib is due to disruption of GH/IGF-1 axis. Pediatr Blood Cancer. (2013) 60:1148–53. doi: 10.1002/pbc.24397, PMID: 23322583

[6] Gupta P Banothu KK Haldar P Gupta AK Meena JP. Effect of imatinib mesylate on growth in pediatric chronic myeloid leukemia: A systematic review and meta-analysis. J Pediatr Hematol Oncol. (2023) 45:227. doi: 10.1097/MPH.0000000000002660, PMID: 37027248

[7] Greyanguly A O’Sullivan S Reid IR Browett P. Imatinib mesylate, increased bone formation, and secondary hyperparathyroidism. New Engl J Med. (2006) 355(23):2494–5. doi: 10.1056/NEJMc062388, PMID: 17151376

[8] Kim TD Schwarz M Nogai H Grille P Westermann J Plöckinger U. Thyroid dysfunction caused by second-generation tyrosine kinase inhibitors in Philadelphia chromosome-positive chronic myeloid leukemia. Thyroid Off J Am Thyroid Assoc. (2010) 20:1209–14. doi: 10.1089/thy.2010.0251, PMID: 20929406

[9] Chang X Zhou L Chen X Xu B Cheng Y Sun S. Impact of imatinib on the fertility of male patients with chronic myelogenous leukaemia in the chronic phase. Target Oncol. (2017) 12:827–32. doi: 10.1007/s11523-017-0521-6, PMID: 28791527

[10] Markovits N Kurnik D Friedrich C Gueta I Halkin H David S. Effects of imatinib on glycemic and lipid profiles: a retrospective cohort study. Leuk Lymphoma. (2022) 63:2224–32. doi: 10.1080/10428194.2022.2068003, PMID: 35475716

[11] Iurlo A Orsi E Cattaneo D Resi V Bucelli C Orofino N. Effects of first- and second-generation tyrosine kinase inhibitor therapy on glucose and lipid metabolism in chronic myeloid leukemia patients: a real clinical problem? Oncotarget. (2015) 6:33944–51. doi: 10.18632/oncotarget.5580, PMID: 26376678 PMC4741815

[12] de Onis M Garza C Victora CG Onyango AW Frongillo EA Martines J. The who multicentre growth reference study: planning, study design, and methodology. Food Nutr Bull. (2004) 25:S15–26. doi: 10.1177/15648265040251S104, PMID: 15069916

[13] Group WMGRS de Onis M. WHO Child Growth Standards based on length/height, weight and age. Acta Paediatr. (2006) 95:76–85. doi: 10.1111/j.1651-2227.2006.tb02378.x, PMID: 16817681

[14] Bidlingmaier M Friedrich N Emeny RT Spranger J Wolthers OD Roswall J. Reference intervals for insulin-like growth factor-1 (igf-i) from birth to senescence: results from a multicenter study using a new automated chemiluminescence IGF-I immunoassay conforming to recent international recommendations. J Clin Endocrinol Metab. (2014) 99:1712–21. doi: 10.1210/jc.2013-3059, PMID: 24606072

[15] De Silva NK Tschirhart J. Puberty-defining normal and understanding abnormal. Curr Treat Options Pediatr. (2016) 2:121–30. doi: 10.1007/s40746-016-0061-9

[16] Lorentzon M Cummings SR. Osteoporosis: the evolution of a diagnosis. J Intern Med. (2015) 277:650–61. doi: 10.1111/joim.12369, PMID: 25832448

[17] Holick MF Binkley NC Bischoff-Ferrari HA Gordon CM Hanley DA Heaney RP. Evaluation, treatment, and prevention of vitamin D deficiency: an Endocrine Society clinical practice guideline. J Clin Endocrinol Metab. (2011) 96:1911–30. doi: 10.1210/jc.2011-0385, PMID: 21646368

[18] American Diabetes Association Professional Practice Committee. 2. Diagnosis and classification of diabetes: standards of care in diabetes—2024. Diabetes Care. (2024) 47:S20–42. doi: 10.2337/dc24-S002, PMID: 38078589 PMC10725812

[19] European Organisation for Research and Treatment of Cancer (EORTC). Quality of Life of Cancer Patients. Brussels, Belgium: EORTC – Quality of Life (2017). Available at: https://qol.eortc.org/questionnaire/eortc-qlq-c30/ (Accessed June 27, 2025).

[20] PedsQL TM. Pediatric Quality of Life Inventory TM. Available online at: https://www.pedsql.org/about_pedsql.html (Accessed June 27, 2025).

[21] Roy Moulik N Keerthivasagam S Pandey A Agiwale J Hegde K Chatterjee G. Treatment and follow-up of children with chronic myeloid leukaemia in chronic phase (CML-CP) in the tyrosine kinase inhibitor (TKI) era—Two decades of experience from the Tata Memorial Hospital paediatric CML (pCML) cohort. Br J Haematol. (2024) 204:1249–61. doi: 10.1111/bjh.19251, PMID: 38098201

[22] Cai Y Liu C Guo Y Chen X Zhang L Chen Y. Long-term safety and efficacy of imatinib in pediatric patients with chronic myeloid leukemia: single-center experience from China. Int J Hematol. (2021) 113:413–21. doi: 10.1007/s12185-020-03042-1, PMID: 33386594

[23] Bansal D Shava U Varma N Trehan A Marwaha RK. Imatinib has adverse effect on growth in children with chronic myeloid leukemia. Pediatr Blood Cancer. (2012) 59:481–4. doi: 10.1002/pbc.23389, PMID: 22052850

[24] Boddu D Thankamony P Guruprasad CS Nair M Rajeswari B Seetharam S. Effect of imatinib on growth in children with chronic myeloid leukemia. Pediatr Hematol Oncol. (2019) 36:189–97. doi: 10.1080/08880018.2019.1610119, PMID: 31298597

[25] Shima H Tokuyama M Tanizawa A Tono C Hamamoto K Muramatsu H. Distinct impact of imatinib on growth at prepubertal and pubertal ages of children with chronic myeloid leukemia. J Pediatr. (2011) 159:676–81. doi: 10.1016/j.jpeds.2011.03.046, PMID: 21592517

[26] Rastogi MV Stork L Druker B Blasdel C Nguyen T Boston BA. Imatinib mesylate causes growth deceleration in pediatric patients with chronic myelogenous leukemia. Pediatr Blood Cancer. (2012) 59:840–5. doi: 10.1002/pbc.24121, PMID: 22378641

[27] Bachrach LK Gordon CM. SECTION ON ENDOCRINOLOGY. Bone densitometry in children and adolescents. Pediatrics. (2016) 138:e20162398. doi: 10.2196/jmir.7004 27669735

[28] Deng M Guan X Wen X Xiao J An X Yu J. Clinical efficacy and safety of imatinib treatment in children and adolescents with chronic myeloid leukemia. Med (Baltimore). (2020) 99:e19150. doi: 10.1097/MD.0000000000019150, PMID: 32049841 PMC7035079

[29] Zheng F Dou X Zhang L Jin J Zhang Y Liu B. Health-related quality of life in children with chronic myeloid leukemia in the chronic phase. J Cancer Res Clin Oncol. (2022) 148:341–50. doi: 10.1007/s00432-021-03832-y, PMID: 34714411 PMC11800997

[30] Samis J Lee P Zimmerman D Arceci RJ Suttorp M Hijiya N. Recognizing endocrinopathies associated with tyrosine kinase inhibitor therapy in children with chronic myelogenous leukemia. Pediatr Blood Cancer. (2016) 63:1332–8. doi: 10.1002/pbc.26028, PMID: 27100618

