# Supplementary material for: Long-term endocrine outcomes and quality of life in paediatric and young chronic myeloid leukaemia patients on tyrosine kinase inhibitor therapy: a prospective study from India
Source: Front Oncol. 2025 Jul 8;15:1598104. doi: 10.3389/fonc.2025.1598104 (PMC12279693; doi:10.3389/fonc.2025.1598104)
Supplement: Supplementary file 1 [file Table1.docx]

**Supplementary table 1**

Normal reference levels of hormones used in the study:

| Growth hormone (ng/ml) | 0·02-1·23 |
| --- | --- |
| Parathyroid hormone (pg/ml) | 15-65 |
| Vitamin D (ng/ml) | <20-deficient  <30-insufficiency |
| 8AM Cortisol (mcg/dl) | 5-23 |
| Adrenocorticotrophic hormone (pg/ml) | 7·2-63 |
| Follicle Stimulating Hormone (mIU/ml) | 1·5-12·4* |
| Luteinizing Hormone (mIU/ml) | 1·7-8·6* |
| Testosterone (ng/ml) | 2·4-8·3 |
| Oestrogen (pg/ml) | 21-251* |
| T4 (mcg/dl)  Thyroid Stimulating Hormone (uIU/ml) | 5-14  0·27-4·2 |
| Low density lipid (mg/dl) | <100-optimal  100-129-near optima/above optimal  130-159-borderline high  160-189-high  ≥190-very high |
| High density lipid (mg/dl) | <40-low  >60-high |
| Very low density lipid (mg/dl) | 0-40 |
| Cholesterol (mg/dl) | <200-desirable  200-239-borderline high  >240-high |
| Triglycerides (mg/dl) | <150-normal  150-199-borderline high  200-499-high  >500-very high |
| Fasting blood glucose level (mg/dl)  OGTT-1hr  OGTT-2hr | 70-110  70-180  70-140 |
| HbA1c (%) | 4·8-5·6-nondiabetic range  5·7-6·4-prediabetic range  >6·5-diabetic range  >7-high risk |

**Varies with menstrual phase; OGTT- oral glucose tolerance test, HbA1c- Glycated haemoglobin*

**Supplementary table 2**

Short stature in patients at <18 years during the time of study

|  | **Patients <18years with short stature (n=8)** |
| --- | --- |
| Height SDS median (range) | -3·0 (-2·0-5·4) |
| Pubertal status at initiation of TKI n (%) | Prepubertal -8 (100·0) |
|  | Post pubertal -0 (0·0) |
| Age at TKI initiation-Mean (SD) | 7·5 (2·9) |
| age at time of study – mean (SD) | 13·1 (2·8) |
| TKI at the time of study n (%) | Imatinib- 8 (100·0) |
| Median duration of TKI in years  Median(range) | 5·5 (3·0-11·0) |
| Patients with low IGF1 levels n (%) | 2 (25·0) |
| Patients with low BMD n (%) | 7 (87·5) |

*SDS- standard deviation score, TKI-tyrosine kinase inhibitors, SD-standard deviation, BMD-bone mineral density*

**Supplementary Table - 3**

|  | **Normal BMI**  **n=38**  n (%) | **Above normal BMI n=12**  n (%) | **Below normal BMI n=21**  n (%) | **P-value** |
| --- | --- | --- | --- | --- |
| **BMI category** | Normal-38 | Overweight-11  Obese-1 | Underweight-21 |  |
| **Pre - Pubertal at initiation of TKI** | 13 (34·2) | 4 (33·3) | 15 (71·4) | P=0.015^†^ |
| **Post Pubertal at initiation of TKI** | 25 (65·8) | 8 (66·7) | 6 (28·6) |  |
| **Age at TKI initiation**  **Mean (SD)** | 12·7 (±3·0) | 12·8 (±4·1) | 9·8 (±4·1) | 0.0012* |
| **Age at time of study –Mean (SD)** | 24·3 (±4·5) | 22·9 (±6·5) | 17·2 (±6·1) | P=0·0013* |
| **TKI at the time of study n (%)** | Imatinib 36 (94·7)  Dasatinib 1 (2·6)  Nilotinib 1 (2·6) | Imatinib 11 (91·6)  Dasatinib 1 (8·3)  Nilotinib 0 (0·0) | Imatinib 17 (81·0)  Dasatinib 4 (19·0)  Nilotinib 0 (0·0) |  |
| **Duration of TKI**  **Median (Range)** | 12 (2·5-19·3) | 10·1 (3·0-19·5) | 6·0 (2·3-22·0) | P=0.0031* |

TKI-tyrosine kinase inhibitors, SD-standard deviation, BMI- body mass index

† Chi-square test for categorical comparisons

*Kruskal–Wallis for continuous variables

**Supplementary Table -4 -Characteristics of patients with abnormalities in BMI, PTH levels and thyroid function**

|  | **Patients with elevated PTH (n=17)#**  **n (%)** | **Patients with Normal PTH**  **(n=53)**  **n (%)** | **Patients with hypothyroidism* (n=7)**  **n (%)** | **Euthyroid**  **(n=64)**  **n (%)** |
| --- | --- | --- | --- | --- |
| Pre - Pubertal at initiation of TKI | 6  (35·3) | 25  (47·2) | 1  (14·3) | 31  (48·4) |
| Post Pubertal at initiation of TKI | 11  (64·7) | 28  (52·8) | 6  (85·7) | 33  (51·6) |
| Age at TKI initiation Mean (SD) | 12·8  (±4·1) | 11·8  (±3·5) | 14·6  (±2·7) | 11·8  (±3·7) |
| Age at time of study –Mean (SD) | 22·7  (±6·3) | 21·8  (±6·1) | 24·6  (±4·9) | 21·6  (±6·1) |
| TKI at the time of study n (%)-Imatinib | 14  (82·4) | 49  (92·5) | 5  (71·4) | 59  (92·2) |
| Dasatinib | 3  (17·6) | 3  (5·7) | 2  (28·6) | 4  (6·3) |
| Nilotinib | 0  (0·0) | 1  (1·9) | 0  (0·0) | 1  (1·6) |
| Duration of TKI  Median (Range) | 7·5  (2·3-19·5) | 10·0  (3·0-22·0) | 12·0  (6·0-18·0) | 8·8  (2·2-22·0) |
|  | Mean z score (SD)-1·1 (1·1)  Vit-D  Deficiency-5 (29·4) | Mean z score (SD)  1·2 (1·0)  Vit-D  Deficiency-17 (32·1) |  |  |

*PTH could not be assessed in one patient.*

** Includes both subclinical(n=6)and clinical(n=1)*

*TKI-tyrosine kinase inhibitors, SD-standard deviation*

*SD – standard deviation, PTH-Para thyroid hormone, TKI-Tyrosine kinase inhibitor.*
